# A Pilot Study Evaluating the Effects of 670 nm Photobiomodulation in Healthy Ageing and Age-Related Macular Degeneration

**DOI:** 10.3390/jcm9041001

**Published:** 2020-04-02

**Authors:** Manjot K. Grewal, Chrishne Sivapathasuntharam, Shruti Chandra, Sarega Gurudas, Victor Chong, Alan Bird, Glen Jeffery, Sobha Sivaprasad

**Affiliations:** 1 Institute of Ophthalmology, University College London, London EC1V 9EL, UK; m.grewal@ucl.ac.uk (M.K.G.); chrishnepriya.sivapathasuntharam.14@ucl.ac.uk (C.S.); shruti.chandra.18@ucl.ac.uk (S.C.); sarega.gurudas.17@ucl.ac.uk (S.G.); alan.bird@ucl.ac.uk (A.B.); g.jeffery@ucl.ac.uk (G.J.); 2 NIHR Moorfields Biomedical Research Centre, Moorfields Eye Hospital, London EC1V 2PD, UK; 3 Eretina Ltd. 8, Spruce Dene, Hazlemere, Bucks HP15 7QF, UK; victor@eretina.org

**Keywords:** photobiomodulation, age-related macular degeneration, 670 nm, dark adaptation, reticular pseudodrusen, subretinal drusenoid deposits, scotopic thresholds, optical coherence tomography

## Abstract

Limited evidence suggests that the application of 670 nm of red light alters the course of aged decline. A previous report on 18 patients showed regression of drusen and improvement in visual functions in patients with intermediate age-related macular degeneration (AMD) by 12 months. We evaluated the functional and structural effects of applying 670 nm light to 31 patients with intermediate AMD and 11 people aged 55 years or above with normal retina. The study eyes were treated daily in the morning with a 670 nm hand-held light source housed in a torch-like tube that emitted energy equivalent to 40 mW/cm^2^ or 4.8J/ cm^2^ for 2 min at the viewing aperture. Visual function in terms of best-corrected visual acuity, low luminance visual acuity, scotopic thresholds and rod-intercept time were compared between baseline and 1, 3, 6 and 12 months. Structural changes on optical coherence tomography OCT and colour photographs were also assessed. Five withdrew consent voluntarily due to the intensity of the study visit assessments and two developed neovascular AMD and were excluded from further treatment and the analysis. In normal ageing, there was an improvement in scotopic thresholds in the group with no AMD by 1.77dB (*p* = 0.03) and no other parameters showed any clinically significant change. In eyes with intermediate AMD, there was no significant improvement in any functional or structural changes at any time point up to 12 months although the compliance was good. This pilot study shows that photobiomodulation with 670 nm has no effect in patients who have already progressed to intermediate AMD.

## 1. Introduction

In healthy ageing and age-related macular degeneration (AMD), there is a progressive loss of rod photoreceptors compared to a minimal decline of the less vulnerable cone photoreceptors [1]. Other changes in the outer retina include thinning and thickening of the retinal pigment epithelium (RPE), increased thickness of the retinal pigment epithelium-Bruch’s membrane complex (RPE-BM complex) and a gradual decline in choriocapillaris [2]. These ageing changes are rarely associated with symptomatic visual function losses. When healthy eyes convert to AMD, the early visible changes are characterized by accumulation of sub-RPE deposits called drusen and in some cases subretinal drusenoid deposits (SDD), otherwise termed reticular pseudodrusen (RPD) [3,4]. None of these signs affect visual acuity but rod-recovery time from a bright flash is delayed and this test can accurately discriminate between healthy ageing of the retina and eyes with AMD [5,6]. Eyes with SDD experience a longer delay in rod-recovery time compared to eyes that do not [6,7,8,9]. These early changes in AMD may progress to the advanced forms of the disease, namely the atrophic type called geographic atrophy and neovascular AMD. The reason why healthy eyes convert to AMD is unknown. With ageing, the membrane potential and function of mitochondria decline, reducing adenosine triphosphate (ATP) production. These changes can signal cell death. As the retina has the greatest energy demand in the body, it is particularly vulnerable to mitochondrial dysfunction [10]. Reduced retinal ATP levels have been reported in murine models and in primates [11,12,13]. Therefore targeting mitochondria may be an option to delay ageing and prevent the switch to AMD or preventing its progression. Recently, photobiomodulation, the application of red to infrared light (= 600–1000 nm), on tissues has been reported to reduce the pace of aged decline. The precise mechanisms of this application are unclear. One concept is that these wavelengths are absorbed by cytochrome c oxidase, the rate-limiting enzyme in mitochondrial respiration, increasing its activity along with mitochondrial membrane potential and ATP production [14,15,16]. We have observed that in aged mice, photobiomodulation reduces inflammation and improves retinal function, as measured by electroretinograms [17,18]. A case series on daily red-light therapy on four patients with non-centre-involving diabetic macular oedema resulted in a reduction in macular fluid [19]. Similarly, clinical investigations have been undertaken to assess the benefit of red light therapy in AMD. A study by Ivandic and Ivandic reported improvement in visual acuity and colour vision lasting up to 36 months, using a low-level laser therapy with a 780 nm wavelength exposure delivered on four occasions (two treatments per week) over a two-week period in patients with dry and wet AMD [20]. In 2017, Merry et al. reported improved visual acuity, contrast sensitivity and regression of drusen in patients with early and intermediate AMD (iAMD) using multi-wavelength LED light therapy (590 nm, 670 nm and 790 nm) with three treatments weekly over a course of three weeks [21]. As visual acuity is not an appropriate outcome measure for early or iAMD, we aimed to establish the possible therapeutic effect of 670 nm light exposure on multiple visual functions and anatomical structures in healthy ageing and AMD with and without SDD to assess whether any of the tests could be reliably used in a future definitive randomized controlled trial on photobiomodulation in AMD.

## 2. Methods

This study was conducted according to the International Conference on Harmonization Guidelines for Good Clinical Practice and the tenets of the Declaration of Helsinki. The study protocol was approved by the National Research Ethics Committee (16/LO/2022). All study participants provided written informed consent.

### 2.1. Study Population

Patients aged over 55 years with either normal retina or iAMD were recruited into this study. iAMD was diagnosed as having at least 1 druse more than 125 μm in diameter, within 1500 μm from the fovea on colour fundus photography based on the Beckman Initiative Scale for AMD classification. The subgroup of patients with a presence of SDD within the iAMD group were defined as those with at least 5 SDDs seen on both in IR imaging and on OCT scans and at least 1 extending to the external limiting membrane (ELM )disrupting the ellipsoid zone. Other inclusion criteria included best-corrected visual acuity of 50 Early Treatment Diabetic Retinopathy Study (EDTRS) letters or better in the study eye, sufficient media clarity, adequate pupillary dilation and fixation to permit quality imaging and psychophysical testing (Grade ≤ 2 on the LOCS III cataract grading scale). Participants also required manual dexterity to use the light device in the correct manner.

Exclusion criteria were neovascular AMD, geographic atrophy, glaucoma or diabetic retinopathy in the study eye, significant systemic disease or history of medication known to affect visual function, epilepsy, history of major ocular surgery in the last 3 months or anticipated within the next 6 months following enrolment in the study eye and any allergies to adhesives or any other component used.

### 2.2. Intervention

The 670 nm light device was a small hand-held light source housed in a 2.5 cm diameter and 8.7 cm long steel tube producing diffuse red light ranging from 650 to 700 nm, emitting energy equivalent to 40 mW/cm^2^ or 4.8 J/cm^2^ (in 120 s) at the viewing aperture. The viewing aperture comprised 9 light-emitting diodes (LED) which were covered by a diffuser to ensure patient comfort and tolerance. The distal end of the device is closed with a push-button switch activated by the application of power, the light source.

### 2.3. Procedure

Participants applied the 670 nm light to the front of the study eye (looking at the red light) for two minutes at a time, every morning for 12 months.

### 2.4. Assessments

All participants had to have assessments of all visual functions and retinal imaging at baseline, 1, 3, 6 and 12 months. A window of ± 2 weeks was allowed. Both rod and cone function tests were done. Visual acuity was assessed using the EDTRS chart at 4 m and low luminance visual acuity was measured using a 2 log neutral density filter. Both tests were recorded in ETDRS letters. Low luminance deficit (LLD) was defined as the difference between best-corrected visual acuity (BCVA) and low-luminance visual acuity (LLVA). Cone function was further assessed by measuring flicker electroretinograms (ERGs) using the handheld RETeval system (LKC Technologies, Inc., Gaithersburg, MD, USA) in both eyes prior to pupillary dilation with adhesive skin electrodes (stimulus strength, 3.0 cd·s/m^2^; frequency, 28.3 Hz). The amplitudes and implicit times were recorded for the waveform component.

The participant was then tested on Medmont Dark-Adapted Chromatic (DAC) perimeter (Medmont International Pty Ltd; Victoria, Australia) for scotopic perimetry after mydriasis and dark adaptation for 40 min. Scotopic thresholds were then measured monocularly in the study eye with a 505 nm (cyan) stimulus at 17 retinal locations, 4°, 8°, 12° eccentricity to the fovea with one added location at 6° inferior in the vertical meridian. Fixation was monitored using an infrared camera built in the perimeter. The light stimulus was 1.73° in size (Goldmann size V) and was presented for 170 ms in a random order across all retinal locations using 3 dB steps. The test was done twice and the average of the two tests was used to calculate the mean retinal sensitivity. The reliability of the tests was also monitored using false positive and false negative assessment and therefore tests exceeding 30% errors in these outputs were excluded from primary analysis.

The participant then underwent tests using the AdaptDx (MacuLogix, Hummelstown, PA, USA), a computer-automated dark adaptometer to measure the rod-recovery time (RIT) after a bright flash. Patients were light adapted in the room for 3–5 min and pupil size was measured (at least 6 mm) before being instructed to place their chin and forehead on the instrument, focusing on the red central light. The test eye was exposed to the equivalent rhodopsin bleach of 82% with the delivery of a 505 nm photoflash subtending 4° and centred at 5° on the inferior vertical meridian (~ 0.80 ms duration). Stimuli were presented for 200 ms using a 3-down/1-up staircase and thresholds were measured until the rod-intercept (time taken to recover 5.0 × 10^−3^ scotopic cd/m^2^ or 3.1 log units of stimulus attenuation) was reached or up to 20 min post-bleach, which ever was shorter. The participant was instructed to press on the response button when light stimuli were seen and had 15 s rest between each threshold measurement. Fixation was monitored using an infrared camera and through the instrument’s fixation error output. When fixation error exceeded 30%, the tests were deemed unreliable and were not included in the primary analysis. A sensitivity analysis including these tests was also done.

### 2.5. Imaging

Fundus photography was then performed with the Topcon TRC-50DX (Topcon Corporation, Tokyo, Japan). The imaging protocol included a 35° stereo pair centred on the fovea and a fundus reflex photograph (anterior segment) to document media opacities. Spectral domain OCT and infrared imaging were then performed with a Spectralis HRA system (Heidelberg Engineering, Heidelberg, Germany). This instrument was used to acquire volume scans (20° × 20°) comprising of 49 parallel OCT B-scans for both eyes. Using the automated real-time (ART) function, 15 images were averaged for each scan. Fundus autofluorescence (FAF) was performed using Spectralis HRA system (Heidelberg Engineering, Heidelberg, Germany). The standard protocol included 30° stereo images centred on the fovea and the disc. Images were acquired once 30 frames were averaged (ART 30).

The colour fundus photographs, OCT and fundus autofluorescence were double graded based on the Beckman Initiative Scale for AMD classification [4]. Each AMD eye was also graded for the presence or absence of SDD. The volumetric analysis over time of the outer nuclear layer (ONL) and retinal pigment epithelium and Bruch’s membrane (RPE-BM) complex within the central 6 mm diameter of the macula was compared between baseline and 12 months and between study groups.

At each study visit, adverse events and compliance were also recorded.

### 2.6. Outcome Measures

As the rod photoreceptors are of the dominant photoreceptor type and use the maximum amount of oxygen in dark adaptation, we measured changes in rod-recovery time after a bright flash as the primary outcome measure. Secondary outcome measures included scotopic thresholds on Medmont (DAC) perimeter, BCVA, LLVA, LLD, amplitude and elicit times on RETeval and structural changes assessing the thickness of the outer nuclear layer and RPE–Bruch’s membrane complex.

### 2.7. Statistical Analysis

Statistical methodology was conservative, without assumption of normality. All statistical analysis and graphs were generated using GraphPad Prism (Version 8.2.1). Linear mixed effects modelling was performed with IBM SPSS Statistics (SPSS Inc. SPSS for Windows, Version 25.0. Chicago, USA). Given the phase I/II nature of the trial, the main analysis comprised of descriptive statistics to summarize the demographic characteristics and outcome measures of healthy ageing and the AMD group. As SDD is a poor prognostic indicator and may suggest more advanced disease, the AMD group was subdivided into those with and without SDD. Change from baseline to 12 months was analysed using paired t-tests (for normally distributed data) or Wilcoxon matched-pairs signed rank test (for non-normally distributed data).

Mixed effects modelling was performed for analysis of repeated measures at each visit (baseline, 1 month, 3 months, 6 months and 12 months) for all functional outcome measures which allows for robust statistical assessment even with missing data. For structural parameters, Multiple group comparative analysis between outcome measures were conducted using the one-way analysis of variance (ANOVA) and *p*-values were adjusted in accordance with the Bonferroni correction.

## 3. Results

### 3.1. Particiapant Characteristics

Of the 42 participants, 12 were included as healthy ageing and 30 had iAMD. SDD were visible in eight eyes with iAMD. One eye with iAMD and also had non-foveal atrophy.35 completed the trial (healthy ageing *n* = 12, AMD subjects *n* = 23, of which 8 also had SDD). Five withdrew consent due to the intensity of the study visit assessments and two developed neovascular AMD and were excluded. Seventeen tests were not included for Adapdx due to fixation errors. The characteristics of each group is shown in Table 1.

### 3.2. Study Outcomes

#### 3.2.1. Primary Outcome

In healthy eyes, rod-intercept time reduced by 3.76 min (*p* = 0.03). No reduction in rod-intercept time was observed in iAMD with no SDD (*p* = 0.49) or iAMD with SDD (*p* > 0.99) groups. A comparison of RIT data was performed with inclusion and exclusion of assessments with more than 30% fixation error; this did not affect the statistical outcome.

#### 3.2.2. Secondary Outcomes

**Figure 1:**
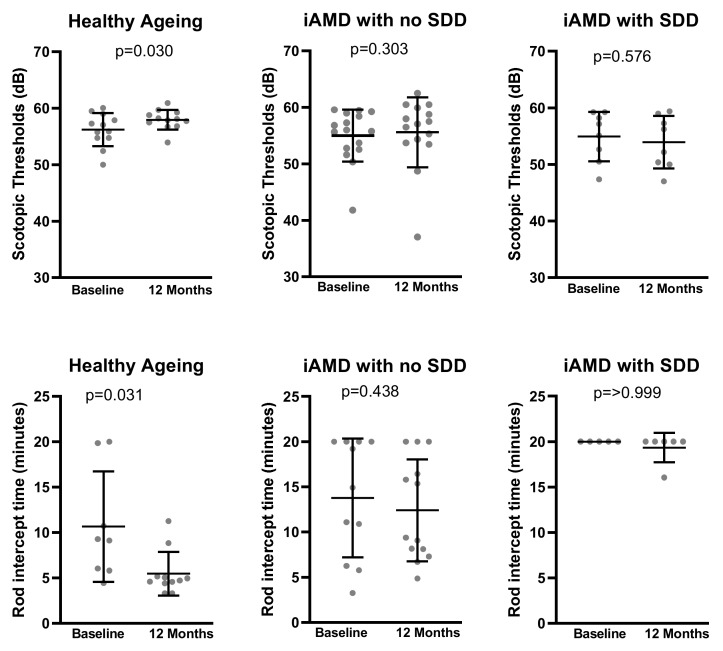
Scatter plots showing change in scotopic thresholds and rod-intercept time (RIT) from baseline to 12 months for 670 nm trial participants. Error bars represent the mean with standard deviation. Only assessments with less than 33% fixation error are displayed in RIT plots. (iAMD = intermediate AMD; SDD = subretinal drusenoid deposits.

The changes from baseline to 12 months for all functional measure outcomes for each study group is summarized in Table 2. The only statistically significant change observed was an improvement in scotopic thresholds in healthy ageing group by 1.77 dB (*p* = 0.03) but there was no change in iAMD with no SDD (*p* = 0.30) or iAMD with SDD (*p* = 0.58) None of the apparent outliers were removed on any plots as they did not affect the statistical outcome of the parameters (Figure 1).

**Figure 2:**
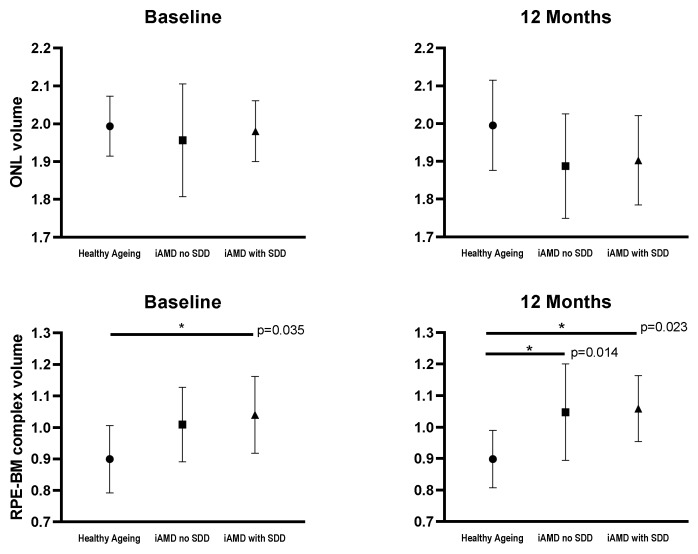
Mean plots showing differences at baseline and 12 months in outer nuclear layer (ONL) and retinal pigment epithelium and Bruch’s membrane complex (RPE-BM) layer volumes between healthy ageing (*n* = 12), iAMD with no SDD (*n* = 16) and iAMD with SDD (*n* = 8) groups. Statistically significant differences are displayed on the plots. Error bars represent SD. iAMD = intermediate AMD; SDD = subretinal drusenoid deposits,

No significant difference was observed in mean ONL volumes between groups at baseline and 12 months (*p* = 0.679, *p* = 0.091 respectively). A substantial variation was found at baseline (*p* = 0.019) and 12 months (*p* = 0.006) between groups in RPE-BM complex mean volumes. This is shown graphically in Figure 2.

### 3.3. Effect of 670 nm Light Therapy Between Groups

We also evaluated the response adjusted for disease groups and time. Table 3, Table 4 and Table 5 shows that there were no statistically significant differences between groups.

### 3.4. Compliance

Compliance was high with 85% of participants declaring their use of the light therapy consistently at the same time every morning with less than five missed days a month.

### 3.5. Adverse Events

There were no device-related serious adverse events in this study. There was no difference between the two groups in the proportion of participants with systemic or ocular (in the study eye) serious adverse events or adverse events unrelated to the progression of late AMD. Two patients in the AMD group progressed to neovascular AMD during the study. These patients presented with asymptomatic subretinal fluid.

## 4. Discussion

This pilot study shows that 670 nm photobiomodulation does not have a positive impact on any of the visual function parameters or outer retinal structures in eyes with iAMD with or without SDD over a period of 12 months. Moreover, there was no difference in changes in visual function and structure between healthy eyes and iAMD. However, in healthy ageing, rod function appears to improve by 12 months with 670 nm light. The scotopic thresholds also increased by 12 months. The rod-recovery time decreased although this is based on a small number of participants who successfully underwent the Adaptdx test in all 4 visits with less than 30% fixation errors.

In AMD eyes, we observed a natural decline in LLVA in keeping with previous natural history reports. In addition, the decreased ONL thickness and the corresponding increase in RPE-BM thickness also suggest disease progression. Our study results are not in agreement with previous results of a prospective study reported by Ivandic and Ivandic and Merry et al. [20,21]. The changes we observed with BCVA more likely represent inter-test variations.

The primary aim of this proof of concept study was to evaluate the effect of 670 nm light on dark adaptometry, a known biomarker of AMD. Interestingly, 670 nm light exposure had no effect on disease groups, but did reduce rod-recovery time in the healthy ageing group. There was a fairly small increase of 1.77 dB in scotopic thresholds in the healthy ageing group at 12 months. A test-retest variability assessment on the Medmont DAC perimeter was performed in our laboratory on young, older healthy and AMD patients (unpublished) and the intersession and intrasession variability or mean difference change was found to be 2.16 dB and 2.42 dB respectively. Although both these rod function test results may be explained by test-retest variations, both these tests showed a positive trend. Therefore, it is worth exploring these tests in a future definitive randomized controlled trial in healthy ageing.

While this trial did not investigate drusen regression due to its naturally dynamic formation and spontaneous regression (or impending geographic atrophy), structural volumetric analysis of the RPE-BM complex layer was assessed to evaluate the effect of 670 nm light therapy. After 12 months, there was no difference from the baseline in drusen volume between the groups. There was also no beneficial impact of 670 nm light exposure on ONL after 12 months. In contrast, a protective effect was described in treated aged CFH mice when compared to controls who had thinner ONL compared to the treated group, although it is appreciated that in humans, the treatment may simply not have been long enough when taking into account the lifespan of a murine model [22]. Also, in Merry et al., trial subjects were given light therapy in both eyes, and this begs the question whether or not there was an additive effect due to the abscopal effect. This implies that the application of the light to a body-part far from the intended point of effect still had an effect [23].

This is the first proof of concept study in discordance with the limited studies published investigating therapeutic modality of photobiomodulation in AMD. The strength of this study was a robust and established assessment protocol of all parameters. There was also a very good overall compliance of more than 85%. The major advantages of the current approach was that these measures were evaluated in the same eyes over a period of time allowing an analysis of the impact of 670 nm on both rods and cones in a well-characterised group of subjects whose fundi were evaluated by a Beckman Initiative Scale classification system. The dosage used in this study was equivalent to 40 mW/cm^2^ or 4.8 J/ cm^2^ (in 120 s) daily for up to 12 months. This was primarily derived from previous successful aged murine retinae and murine models of AMD studies [11,12,17,24]. It was also in alignment with previously published clinical AMD trials which used photobiomodulation regimen of 780 nm semiconductor laser delivered twice a week for 2 weeks for 40 s at 0.3 J/cm^2^ [20] and at 670 nm, for 88 s, delivering 4–7.7 J/cm^2^ during a total of 18 treatments (three times a week for six weeks) yielding positive outcomes [21].

Some limitations of this study are the lack of a control arm. Eight eyes in the AMD group had SDD. Although little is known about the pathogenesis of SDD, it is a surrogate marker of significant rod function decline, poor prognosis and disease progression. Due to the heterogeneity in the AMD population and the small sample size, we may have recruited a group of eyes that had no further potential for improvement. Another limitation was the lack of a mechanism to verify whether the device light output remained constant over the trial. There are very limited clinical studies on 670 nm photobiomodulation and the dosing of the light therapy was based on the study by Merry et al. and the animal studies, which again may not be appropriate based on these study results.

These study results are contrary to those observed by Merry et al. who evaluated three wavelengths of light, 590 nm, 670 nm and 790 nm simultaneously in eyes with early and intermediate AMD (AREDS 2–4). Both 670 and 790 nm are red and infra-red wavelengths and so the effects are likely to be complimentary. The exact effect of 590 nm light on the retina is unclear and there is no obvious reason for its use. Unlike our study results, the changes in visual function were also observed from as early as three weeks and structural changes in three months, indicating the need for a randomized controlled trial to ensure that the study results are clinically meaningful and are not confounded by test-retest variability.

As the therapy works widely in animal models including normal ageing and induced pathology, it is likely that the window of treatment opportunity, dosing and timing of intervention need to be investigated further. It is possible that there may be an age-dependent time frame where rods can be rescued to enhance scotopic thresholds. As the therapy is suggestive of rod function improvement in healthy ageing, further research is required to establish the optimal age window that would be benefit from treatment.

## Figures and Tables

**Table 1:** Participant characteristics in each group at baseline visit who completed 12 month study duration.

	Healthy Ageing (*n* = 12)	iAMD with no SDD (*n* = 15)	iAMD with SDD (*n* = 8)
Mean Age (± SD)	69.9 (± 2.5)	69.4 (± 7.2)	69.3 (± 6.8)
Ethnicity (*n*, %)			
Asian	1 (8%)	1 (7%)	0 (0%)
Caucasian	11 (92%)	15 (93%)	8 (100%)
Gender balance			
*n* (% female)	7 (58%)	10 (67%)	5 (63%)
Mean BCVA	87.1 ± 6.5	84.8 ± 7.6	81.6 ± 3.7
(ETDRS letters ± SD)			
Smoking status			
Current or former smoker (%)	33%	60%	25%
Diabetes history (%)	0%	0%	0%
Hypertension history (%)	33%	20%	38%
Hyperlipedemia history (%)	50%	26%	13%
AREDS or other eye supplements	0%	60%	63%

iAMD = intermediate AMD; SDD = subretinal drusenoid deposits; BCVA = Best Corrected Visual Acuity; ETDRS = Early Treatment Diabetic Retinopathy Study; AREDS = Age-Related Eye Disease Study.

**Table 2:** Twelve-month change from baseline in functional outcome measures for each study group.

Outcomes	Healthy Ageing Mean (SD); *n*			iAMD with no SDD Mean (SD); *n*			iAMD with SDD Mean (SD); *n*		
	Baseline	12 Months	*p* Value	Baseline	12 Months	*p* Value	Baseline	12 Months	*p* Value
BCVA (ETDRS letters)	87.1 (6.5); 12	85.7 (6.4); 12	0.356	84.8 (7.6); 15	85.7 (6.6); 15	0.456	81.6 (3.7); 8	83.0 (3.9); 8	0.211
LLVA (ETDRS letters)	73.5 (6.0); 12	70.4 (6.2); 12	0.182	70.8 (8.1); 15	67.2 (9.1); 15	0.005	68.1 (6.3); 8	65.9 (8.7); 8	0.188
LLD (ETDRS letters)	13.6 (3.7); 12	15.3 (4.3); 12	0.363	14.0 (5.5); 15	18.5 (6.3); 15	0.006	13.5 (5.9); 8	17.1 (8.3); 8	0.050
Scotopic Thresholds (dB)	56.18 (2.94); 12	57.96 (1.75); 12	0.030	54.69 (4.59); 15	55.58 (6.20); 15	0.303	54.92 (4.39); 8	53.93 (4.64); 8	0.576
RIT (minutes)	9.35 (5.21); 7	5.59 (2.58); 7	0.031	14.92 (6.12); 8	13.24 (5.17); 8	0.438	20.00 (0.00); 5	19.21 (1.77); 5	>0.999
Photopic 28.3Hz flicker ERG amplitude (µV)	14.21 (5.92); 11	11.00 (4.21); 11	0.020	16.82 (7.74); 12	14.54 (6.30); 12	0.583	14.59 (4.67); 7	12.43 (5.88); 7	0.443
Photopic 28.3Hz flicker ERG timing (ms)	27.80 (1.28); 11	28.05 (0.82); 11	0.338	28.31 (1.34); 12	28.88 (1.28); 12	0.192	28.13 (1.23); 7	29.43 (1.14); 7	0.038

Means alongside the standard deviation (bracketed) values are shown. The *p*-values specified relate to the significance of the difference in mean change within study groups. iAMD = intermediate AMD; SDD = subretinal drusenoid deposits; BCVA = Best Corrected Visual Acuity; ETDRS = Early Treatment Diabetic Retinopathy Study; LLVA =l ow luminance visual acuity; LLD = low luminance deficit; RIT = rod intercept time; ERG = electroretinogram.

**Table 3:** Change in visual acuity outcomes; best-corrected visual acuity (BCVA), low-luminance visual acuity (LLVA) and low-luminance deficit (LLD).

BCVA (ETDRS Letters)	Mean (SD); *n*			Change from Baseline Mean (SE)			Adjusted Difference Between Groups (95% CI)			
	Healthy ageing	iAMD with no SDD	iAMD with SDD	Healthy ageing	iAMD with no SDD	iAMD with SDD	iAMD with no SDD vs HA	*p*-value	iAMD- with SDD vs HA	*p*-value
Baseline	87.1 (6.5); 12	84.8 (7.6); 15	81.6 (3.7); 8	-	-	-	-	-	-	-
1 Month	87.4 (7.2); 12	85.9 (6.4); 15	84.1 (4.5); 8	0.33 (1.05)	1.13 (0.93)	2.50 (1.28)	0.80 (−1.97–3.58)	0.569	2.17 (−1.10–5.43)	0.192
3 Months	86.2 (8.8); 12	86.0 (5.5); 15	83.1 (5.6); 7	−0.92 (1.05)	1.20 (0.93)	1.42 (1.34)	2.12 (−0.66–4.89)	0.134	2.34 (−1.02–5.70)	0.170
6 Months	86.5 (6.3); 10	84.8 (7.3); 13	84.2 (3.7); 6	0.57 (1.11)	0.89 (0.98)	1.40 (1.41)	0.32 (−2.58–3.22)	0.829	0.84 (−2.69–4.37)	0.640
12 Months	85.7 (6.4); 12	85.7 (6.6); 15	83.0 (3.9); 8	−1.42 (1.05)	0.87 (0.93)	1.38 (1.28)	2.28 (−0.47–5.03)	0.104	2.79 (−0.47–6.05)	0.092
**LLVA** **(ETDRS Letters)**	**Mean (SD); *n***			**Change from baseline Mean (SE)**			**Adjusted difference between groups** **(95% CI)**			
	Healthy ageing	iAMD with no SDD	iAMD with SDD	Healthy ageing	iAMD with no SDD	iAMD with SDD	iAMD with no SDD vs HA	*p*-value	iAMD- with SDD vs HA	*p*-value
Baseline	73.5 (6.0); 12	70.8 (8.1); 15	68.1 (6.3); 8	-	-	-	-		-	-
1 Month	71.7 (7.0); 12	68.6 (8.2); 15	68.9 (6.5); 8	−1.83 (1.50)	−2.20 (1.34)	0.75 (1.84)	−0.37 (−4.35–3.62)	0.856	2.58 (−2.12–7.28)	0.279
3 Months	74.3 (7.2); 12	70.3 (8.0); 15	69.4 (7.7); 7	0.75 (1.50)	−0.53 (1.34)	0.90 (1.92)	−1.28 (−5.27–2.70)	0.525	0.15 (−4.69–4.98)	0.950
6 Months	75.0 (6.1); 10	69.5 (8.9); 13	69.7 (8.4); 6	2.25 (1.59)	−0.05 (1.40)	2.05 (2.02)	−2.30 (−6.50–1.90)	0.281	−0.20 (−5.29–4.89)	0.939
12 Months	70.4 (6.2); 12	67.2 (9.0); 15	65.9 (8.7); 8	−3.08 (1.50)	−3.60 (1.34)	−2.25 (1.84)	−0.52 (−4.50–3.47)	0.798	0.83 (−3.87–5.53)	0.726
**LLD** **(ETDRS Letters)**	**Mean (SD); *n***			**Change from baseline Mean (SE)**			**Adjusted difference between groups** **(95% CI)**			
	Healthy ageing	iAMD with no SDD	iAMD with SDD	Healthy ageing	iAMD with no SDD	iAMD with SDD	iAMD with no SDD vs HA	p-value	iAMD with SDD vs HA	P-value
Baseline	13.6 (3.7); 12	14.0 (5.5); 15	13.5 (5.9); 8	-	-	-	-		-	-
1 Month	15.6 (5.8); 12	17.3 (7.1); 15	15.3 (5.0); 8	2.17 (1.57)	3.33 (1.40)	1.75 (1.92)	1.17 (−2.99–5.32)	0.580	−0.42 (−5.32–4.49)	0.867
3 Months	11.9 (5.7); 12	15.7 (7.0); 15	13.7 (5.6); 7	−1.67 (1.57)	1.73 (1.40)	2.13 (1.92)	3.40 (−0.76–7.56)	0.108	3.79 (−1.11–8.69)	0.128
6 Months	11.5 (3.9); 10	15.3 (7.3); 13	14.5 (8.9); 6	−1.74 (1.66)	0.94 (1.46)	−0.62 (2.10)	2.68 (−1.70–7.06)	0.229	1.12 (−4.19–6.42)	0.678
12 Months	15.3 (4.3); 12	18.5 (6.3); 15	17.1 (8.3); 8	1.67 (1.57)	4.47 (1.40)	3.63 (1.92)	2.80 (−1.36–6.96)	0.185	1.96 (−2.94–6.86)	0.431

iAMD = intermediate AMD; SDD = subretinal drusenoid deposits; HA=healthy ageing; BCVA = Best Corrected Visual Acuity; ETDRS = Early Treatment Diabetic Retinopathy Study; LLVA =low luminance visual acuity; LLD=low luminance deficit.

**Table 4:** Change in rod function outcome measures; scotopic threshold and rod-intercept time (RIT).

Rod-Intercept Time (RIT, minutes)	Mean (SD); *n*		Change from Baseline Mean (SE)			Adjusted Difference Between Groups (95% CI)				
	Healthy ageing	iAMD with no SDD	iAMD with SDD	Healthy ageing	iAMD with no SDD	iAMD with SDD	iAMD with no SDD vs HA	*p*-value	iAMD with SDD vs HA	*p*-value
Baseline	10.67 (6.09); 8	14.52 (6.41); 10	20.00 (0.00); 5	-	-	-	-	-	-	-
1 Month	9.06 (5.33); 8	14.19 (6.08); 12	17.29 (4.36); 5	−1.89 (1.72)	0.93 (1.55)	−0.42 (2.45)	2.82 (−1.78–7.42)	0.227	1.47 (−4.47–7.40)	0.625
3 Months	5.83 (3.33); 11	12.51 (6.89); 15	19.92 (0.22); 7	−5.61 (1.74)	−0.62 (1.50)	−0.27 (2.40)	4.98 (0.43–9.54)	0.032	5.33 (−0.55–11.21)	0.075
6 Months	5.39 (2.60); 10	13.27 (6.42); 13	17.21 (4.36); 5	−6.37 (1.77)	−0.44 (1.57)	−3.93 (2.45)	5.92 (1.24–10.61)	0.014	2.44 (−3.56–8.43)	0.422
12 Months	5.48 (2.40); 11	12.40 (5.64); 13	19.34 (1.61); 6	−5.80 (1.74)	−1.20 (1.57)	−0.46 (2.40)	4.60 (−0.04–9.25)	0.052	5.34 (−0.54–11.23)	0.075
**Scotopic Thresholds (dB)**	**Mean (SD); *n***			**Change from baseline Mean (SE)**			**Adjusted difference between groups** **(95% CI)**			
	Healthy ageing	iAMD with no SDD	iAMD with SDD	Healthy ageing	iAMD with no SDD	iAMD with SDD	iAMD with no SDD vs HA	*p*-value	iAMD with SDD vs HA	*p*-value
Baseline	56.18 (2.94); 12	54.70 (4.60); 15	54.92 (4.40); 8	-	-	-	-		-	-
1 Month	55.27 (3.23); 12	56.05 (4.01); 15	54.85 (5.42); 8	−0.91 (1.09)	1.36 (0.98)	−0.07 (1.34)	2.27 (−0.63–5.18)	0.124	0.84 (−2.58–4.27)	0.628
3 Months	57.47 (2.32); 12	57.46 (3.10); 15	55.58 (4.15); 7	1.29 (1.09)	2.77 (0.98)	0.83 (1.40)	1.48 (−1.42–4.39)	0.314	−0.46 (−3.97–3.06)	0.797
6 Months	57.42 (1.93); 10	56.11 (3.73); 13	54.84 (3.39); 6	1.21 (1.16)	1.89 (1.02)	0.09 (1.47)	0.67 (−2.39–3.73)	0.664	−1.12 (−4.82–2.58)	0.550
12 Months	57.95 (1.75); 12	55.58 (6.20); 15	53.93 (4.64); 8	1.77 (1.09)	0.89 (0.98)	−0.99 (1.34)	−0.87 (−3.78–2.03)	0.553	−2.76 (−6.18–0.67)	0.114

iAMD = intermediate AMD; SDD = subretinal drusenoid deposits; HA=healthy ageing.

**Table 5:** Change in cone function outcome measures; photopic 28.3Hz flicker ERGs amplitude and timing.

Photopic Flicker Timing (ms)	Mean (SD); *n*			Change from Baseline Mean (SE)			Adjusted Difference between Groups (95% CI)			
	Healthy ageing	iAMD with no SDD	iAMD with SDD	Healthy ageing	iAMD with no SDD	iAMD with SDD	iAMD with no SDD vs HA	*p*-value	iAMDwith SDD vs HA	*p*-value
Baseline	27.80 (1.28); 11	28.33 (1.29); 13	28.20 (1.16); 8	-	-	-	-	-	-	-
1 Month	27.89 (1.00); 11	28.44 (0.92); 14	28.74 (1.37); 7	0.03 (0.29)	−0.20 (0.27)	0.13 (0.35)	−0.22 (−1.01–0.56)	0.569	0.10 (−0.80–1.00)	0.827
3 Months	27.63 (1.50); 12	28.22 (0.78); 14	28.70 (0.95); 7	−0.30 (0.28)	−0.20 (0.26)	−0.09 (0.35)	0.09 (−0.67–0.86)	0.805	0.20 (−0.69–1.09)	0.649
6 Months	27.81 (0.97); 10	28.59 (1.09); 12	28.23 (0.99); 6	−0.20 (0.30)	0.22 (0.27)	−0.16 (0.37)	0.42 (−0.38–1.22)	0.302	0.04 (−0.90–0.98)	0.937
12 Months	27.99 (0.82); 12	29.01 (1.22); 14	29.43 (1.14); 7	0.06 (0.28)	0.55 (0.26)	0.86 (0.35)	0.48 (−0.28–1.24)	0.210	0.80 (−0.09–1.69)	0.077
**Photopic flicker Amplitude (µV)**	**Mean (SD); *n***			**Change from baseline Mean (SE)**			**Adjusted difference between groups** **(95% CI)**			
	Healthy ageing	iAMD with no SDD	iAMD with SDD	Healthy ageing	iAMD with no SDD	iAMD with SDD	iAMD with no SDD vs HA	p-value	iAMD with SDD vs HA	P-value
Baseline	14.21 (5.92); 11	16.75 (7.41); 13	14.24 (4.44); 8	-	-	-	-	-	-	-
1 Month	15.04 (7.86); 11	15.64 (7.36); 14	17.59 (4.77); 7	1.18 (1.65)	−0.08 (1.53)	2.64 (1.99)	−1.26 (−5.72–3.10)	0.575	1.46 (−3.66–6.59)	0.573
3 Months	12.39 (7.74); 12	15.42 (5.47); 14	14.16 (3.64); 7	−1.61 (1.60)	0.72 (1.49)	−0.19 (1.99)	2.33 (−2.01–6.68)	0.289	1.42 (−3.65–6.49)	0.580
6 Months	12.77 (4.84); 10	14.63 (5.66); 12	14.40 (5.98); 6	−0.29 (1.70)	−0.90 (1.55)	−0.29 (2.10)	−0.61 (−5.17–3.95)	0.791	0.00 (−5.35–5.35)	1.000
12 Months	10.88 (4.03); 12	14.32 (5.91); 14	12.43 (5.88); 7	−3.12 (1.60)	−1.21 (1.50)	−1.87 (2.00)	1.91 (−2.43–6.26)	0.385	1.25 (−3.82–6.33)	0.626

iAMD = intermediate AMD; SDD = subretinal drusenoid deposits; HA=healthy ageing
